# Genome-wide analysis of *TPX2* gene family in *Populus trichocarpa* and its specific response genes under various abiotic stresses

**DOI:** 10.3389/fpls.2023.1159181

**Published:** 2023-03-13

**Authors:** Meng Qi, Shengjie Wang, Na Li, Lingfeng Li, Yue Zhang, Jingyi Xue, Jingyi Wang, Rongling Wu, Na Lian

**Affiliations:** ^1^ State Key Laboratory of Tree Genetics and Breeding, College of Biological Sciences and Technology, Beijing Forestry University, Beijing, China; ^2^ National Engineering Research Center of Tree Breeding and Ecological Restoration, Beijing Forestry University, Beijing, China

**Keywords:** *Populus*, gene expression pattern, gene structure, phase separation, stress treatment, *PtTPX2* gene family

## Abstract

Microtubules are essential for regulating cell morphogenesis, plant growth, and the response of plants to abiotic stresses. TPX2 proteins are the main players determining the spatiotemporally dynamic nature of the MTs. However, how TPX2 members respond to abiotic stresses in poplar remains largely unknown. Herein, 19 TPX2 family members were identified from the poplar genome and analyzed the structural characteristics as well as gene expression patterns. All TPX2 members had the conserved structural characteristics, but exhibited different expression profiles in different tissues, indicating their varying roles during plant growth. Additionally, several light, hormone, and abiotic stress responsive *cis*-acting regulatory elements were detected on the promoters of *PtTPX2* genes. Furthermore, expression analysis in various tissues of *Populus trichocarpa* showed that the *PtTPX2* genes responded differently to heat, drought and salt stress. In summary, these results provide a comprehensive analysis for the *TPX2* gene family in poplar and an effective contribution to revealing the mechanisms of PtTPX2 in the regulatory network of abiotic stress.

## 1 Introduction

Microtubules (MTs) are hollow tubular structures polymerized by the α-tubulin and β-tubulin heterodimers that play critical roles in cell division, cell expansion and morphogenesis during plant growth and cell morphogenesis (Fletcher and Mullins, 2010; Ren et al., 2017). The functions and dynamics of MTs depend on multiple microtubule-associated proteins (MAPs) and other regulators, such as γ-tubulin complex (γ-TuC), microtubule bundling proteins and microtubule destabilizing proteins (Lloyd, 2011; Hamada, 2014). It has been reported that MTs can rapidly alter their organization in response to various environmental signals, such as light, low temperatures and salt stress (Chen et al., 2016; Lian et al., 2021). For instance, salt stress induces the rapid depolymerization of MTs, followed by the reassembly of new microtubule networks, which is vital for plant survival under high‐salinity environment (Wang et al., 2011; Wang and Mao, 2019). Furthermore, several MAPs have been implicated in these processes. For example, the degradation of SPIRAL1, a microtubule-stabilizing protein, is required for the salinity-induced rapid depolymerization of MTs and for plant salt-stress tolerance (Wang et al., 2011).

Targeting Protein for Xklp2 (TPX2) contains a highly conserved TPX2 domain (Pfam: PF06886) and acts as a microtubule-binding protein to regulate the stability and organization of cortical MTs in animals and plants (Wittmann et al., 2000; Brunet et al., 2004; Vos et al., 2008). TPX2 family proteins contain TPX2, TPX2-importin, TPX2-C^KLEEK^ and WAVE-DAMPENED2-like New Domain (WAND). TPX2_importin domain mediates the localization of Xklp2 to the mitotic spindle. The TPX2-C domain, a highly conserved and kinesin-interacting domain, is located on the very C-terminus. Plant TPXL binds and activates Aurora 1 through the conserved Aurora-binding domain on the N-terminus, and Aurora 1 phosphorylates TPX2 independently of the binding domain (Smertenko et al., 2021). In addition, WAND, a class of conservative domain, is also discovered. Furthermore, the *Xenopus* TPX2 protein undergoes phase separation to form droplets with tubulin, which is essential for microtubule nucleation. The disordered N-terminal region of TPX2 enhances the phase separation and efficiency of MTs nucleation (King and Petry, 2020; Safari et al., 2021).

In the past two decades, several TPX2 proteins have been reported in various plants during growth and development, such as TPX2, MAP20, WVD2 and WDL (Perrin et al., 2007; Rajangam et al., 2008; Liu et al., 2013; Sun et al., 2015; Lian et al., 2017; Smertenko et al., 2020). The *Arabidopsis thaliana* WVD2 (AtWVD2) promotes MTs bundling to improve their stability, which plays a critical role in polar cell elongation (Perrin et al., 2007). AtWDL3 stabilizes MTs and functions in hypocotyl cell elongation in response to light, while the protein is degraded under dark conditions (Liu et al., 2013; Lian et al., 2017). AtWDL5 stabilizes MTs to promote ethylene-associated MTs reassembly (Sun et al., 2015). *Populus tremula* × *tremuloides* MAP20 (PttMAP20) stabilizes MTs and acts as a target of the cell wall synthesis herbicide 2,6-dichlorobenzonitrile, playing an important role in cell wall biosynthesis (Rajangam et al., 2008). The *Brachypodium distachyon* MAP20 (BdMAP20) suppresses MTs depolymerization and functions in metaxylem pit development (Smertenko et al., 2020). Furthermore, TPX2 proteins are also involved in various abiotic stress responses in plants (Dou et al., 2018; Smertenko et al., 2020). AtWDL5 is involved in ethylene‐mediated MTs reassembly in response to salt stress (Dou et al., 2018). BdMAP20 is involved in the regulation of vascular bundle development and contributes to drought recovery (Smertenko et al., 2020).

Poplar is an economically and ecologically important perennial woody plant that is widely cultivated. It is seriously affected by various environmental stresses such as soil salinization, heat and drought (Zhang et al., 2019). Poplar also serves as a model for elucidating physiological and molecular mechanisms of development and stress tolerance in tree species based on its rapid growth characteristics (Wu et al., 2021). Research has shown that MAPs are key regulatory molecules participating in plant growth and responses to environmental signals. At present, most studies on TPX2 proteins in abiotic stress still focus on model plants, such as *Arabidopsis*. In poplars, the family members of TPX2 and their regulatory mechanisms in response to abiotic stress are largely unknown. In this study, *Populus trichocarpa TPX2* (*PtTPX2*) genes were systematically and comprehensively analyzed. Specifically, analyses of phylogenetic relationships, protein physicochemical properties, gene structures, protein domain, chromosome distributions, intrinsically disordered protein regions, promoter *cis*-acting elements of *PtTPX2* genes and collinearity analysis of species were performed. RT-qPCR showed that *PtTPX2* genes have different expression patterns in poplar. By analyzing the results of RT-qPCR, it was found that *PtWDL6* was up-regulated in response to salt, heat and drought treatments. These findings indicate that *PtWDL6* plays an important role in the resistance to abiotic stresses. Therefore, we cloned the *PtWDL6* gene and found that PtWDL6 can bind MTs through subcellular localization analysis. These results suggest that *PtWDL6* may be involved in poplar response to abiotic stress by regulating MTs. Collectively, this study performs an initial characterization of the structures and functions of the *PtTPX2* family, extends the relationship between the plant *TPX2* family and abiotic stress tolerance and establishes a foundation for future studies on the role of *TPX2* genes in stress tolerance.

## 2 Materials and methods

### 2.1 Plant materials and treatments

One-month-old *Populus alba × Populus glandulosa* (84K) seedlings were cultivated on sterile WPM at 25°C with 16 h of light of 5,000 lx and 8 h of dark. For salt stress, one-month-old seedlings were transferred from initial solid WPM medium (without NaCl) to liquid WPM media supplemented with 200 mM NaCl for 24 hours (Xu et al., 2018). For heat stress, one-month-old seedlings were transferred to 42 °C for 24 h. For drought stress, one-month-old seedlings were transferred to a medium containing 200 mM mannitol (simulated drought) for 24 h. Controls for the above treatments were incubated without any treatment. After treatment, various tissues were harvested from three plants of each treatment in different times. All samples obtained were frozen in liquid nitrogen and rapidly stored at - 80°C for subsequent experiments.

### 2.2 Identification and phylogenetic analysis of TPX2 genes in *Poplus trichocarpa*

To identify putative *TPX2* genes in *Populus trichocarpa*, the protein sequence of *Arabidopsis thaliana TPX2* genes were obtained from TAIR (https://www.arabidopsis.org/, accessed on 20 October 2022) and used as the query sequences to screen all the candidate proteins in *Populus trichocarpa* with the BLASTp search (https://phytozome-next.jgi.doe.gov/, accessed on 20 October 2022) (Luo et al., 2022). In order to ensure the accuracy and comprehensiveness of the sequences, the Pfam number (PF06886) and HMM model files of the TPX2 conserved domain were searched through the PFAM database (http://pfam.xfam.org/, accessed on 20 October 2022), and the *TPX2* family genes were screened by the HMM model file on the HMMER website (http://hmmer.org/, accessed on 20 October 2022) (Cao et al., 2020).

The amino acid sequences of TPX2 proteins from *Arabidopsis thaliana*, *Populus trichocarpa*, *Gossypium hirsutum* and *Eucalyptus grandis* were obtained from TAIR (https://www.arabidopsis.org/, accessed on 25 December 2022) and Phytozome (https://phytozome-next.jgi.doe.gov/, accessed on 25 December 2022). These TPX2 sequences were aligned using the ClustalW program (Chenna et al., 2003). The parameters were set by default. A neighbor-joining (NJ) phylogenetic tree was constructed using the MEGA7.0.26 program with 1000 bootstrap replicates (Lei et al., 2019).

### 2.3 Characteristic analysis of PtTPX2

The length of the coding sequences and number of amino acids were calculated by using the TBtools software (Chen et al., 2020). The molecular weight and isoelectric point of PtTPX2 proteins were analyzed by using the ExPASy ProtParam tool (https://web.expasy.Org/protparam/, accessed on 20 October 2022) (Tuskan et al., 2006). The conserved motifs of PtTPX2 proteins were predicted by the online website MEME (http://meme-suite.org/index.html/, accessed on 20 October 2022) (Bailey and Gribskov, 1998). To further visualize the motifs’ compositions and protein structures, integration analysis was conducted using TBtools software. Based on the General Feature Format (GFF) information from the Phytozome database, the chromosomal distribution of the *PtTPX2* genes was con-firmed and visualized by using Mapchart software (Voorrips, 2002). The promoter sequences 2000 bp upstream of the *Populus trichocarpa TPX2* genes were extracted from the Phytozome database. The prediction of *cis*-acting elements of the promoter region was identified using the PlantCare database (http://bioinformatics.psb.ugent.be/webtools/plantcare/html/, accessed on 20 October 2022) (Lescot et al., 2002).

### 2.4 Chromosomal distribution and collinear analysis of PtTPX2

The chromosomal positions of genes were mapped using the TBtools software (Chen et al., 2020) based on annotated files of genomic structural information of *Arabidopsis thaliana*, *Populus trichocarpa*, *Gossypium hirsutum* and *Eucalyptus grandis*. These genomic data were obtained from Phytozome (https://phytozome-next.jgi.doe.gov/, accessed on 25 January 2023). TBtools software was used to visualize intraspecific and interspecific collinearity of *Populus trichocarpa*. Advanced Circos function from TBtools software was used for visualization within *Populus trichocarpa* species. Multiple Synteny Plot function from TBtools software was used to visualize collinearity of *Populus trichocarpa* and *Arabidopsis thaliana*, *Populus trichocarpa* and *Gossypium hirsutum*, *Populus trichocarpa* and *Eucalyptus grandis*.

### 2.5 Prediction of PtTPX2 proteins’ intrinsically disordered regions

Intrinsically disordered regions (IDRs) are protein regions characterized by lack of definite structures. IDRs of all PtTPX2 proteins were predicted by using the online PONDR program (http://www.pondr.com/, accessed on 25 January 2023) (Xue et al., 2010). The presence of IDRs makes it easier for proteins to form droplet shape, inducing phase transition generation and regulation.

### 2.6 Total RNA extraction and cDNA reverse transcription synthesis

Total RNA was extracted using the Eastep Super Total RNA Extraction Kit (LS1040, Shanghai Promega, Shanghai, China). The quality of RNA samples were determined by the NanoDrop 8000 (Thermo Fisher Scientific, Waltham, Massachusetts, USA) (Desjardins and Conklin, 2010; Lei et al., 2019). Then, using 1 µg RNA as the template, the RNA was reverse transcribed into cDNA using the rapid quantitative RT Supermix Kit (M-MLV Reverse Transcriptase, Mi Si Century Biotechnology company, Shanghai, China) (Qi et al., 2022). The cDNA was stored at - 20 °C for subsequent RT-qPCR experiments.

### 2.7 PCR primer design and real-time quantitative PCR analysis

Specific RT-qPCR primers were designed using Primer 3 plus software (https://www.primer3plus.com/index.html/, accessed on 1 August 2022), and then the best primers for specificity were selected (**Supplementary Table S1**). The number of primer bases was between 18 and 20 bp, and the sequence length was between 80 and 120 bp. Poplar *18S rRNA* was used as a reference gene to normalize the expression data. RT-qPCR was detected by CFX connect in a 10 µl per well system. Calculations were performed using the 2^-△△Ct^ method with a *t*-test to analyze significant differences (Meng et al., 2016). Three biological replicates were set up for each sample and three technical replicates for each biological replicate. Microsoft Excel software was used for data analysis, Graphpad prism9 and TBtools software was used for drawing.

### 2.8 Subcellular localization of PtWDL6 protein

The amplified CDS of *PtWDL6* and the sequence of expressing green fluorescent protein (GFP) were inserted into the plasmid vector (pCAMBIA1390) by homologous recombination. Subcellular localization of PtWDL6 and cortical MTs was visualized using transiently expressed *UBQ : PtWDL6-GFP* and *UBQ : MBD-mCherry* constructs expressed in tobacco leaf epidermal cells (Liu et al., 2013; Dou et al., 2021). The plasmid was transformed into *Agrobacterium tumefaciens* GV3101, and the two vectors were transiently co-expressed in tobacco epidermal cells. All fluorescence signals of the samples were detected using a confocal laser scanning microscope system (Zeiss LSM 810).

## 3 Results

### 3.1 Characterization and phylogenetic analysis of PtTPX2 genes

In this study, we screened the *TPX2* genes from the *Populus trichocarpa v4.1* (https://phytozome-next.jgi.doe.gov, accessed on 21 October 2022), named as *PtMAP20*, *PtMAP20L*, *PtTPX2* and *PtWDL*. Detailed characteristics and information of these genes were analyzed and summarized in **Table 1**, including the molecular weight (Mw), the isoelectric point (PI) and the number of amino acids (AAs). In this family, the proteins ranged from 177 (PtMAP20) to 817 (PtTPX2-1) amino acids in length. The predicted molecular weight varied from 20.79 kDa (PtMAP20) to 92.33 kDa (PtTPX2-1). Except for PtWDL10, the number of predicted isoelectric points was more than seven, indicating that PtTPX2 proteins are rich in basic amino acids. Amino acid composition analysis showed that the proportion of amino acid composition of PtTPX2 proteins were similar. The proportion of the charged amino acids was 36.01% to 45.63%. The proportion of the acidic amino acids was between 12.57% and 17.48%, and the proportion of basic amino acids was between 18.79% and 23.07%. The proportion of polar amino acids ranged from 22.72% to 32.61%, and the proportion of hydrophobic amino acids ranged from 20.6% to 26.83%.

**Table 1:** Information of PtTPX2 protein structures and amino acid ratios.

Amino acid (s)	Gene ID	Mw	PI	AAs	Number count (% by weight)				
					Charged	Acidic	Basic	Polar	Hydrophobic
					(R K H Y C D E)	(D E)	(K R)	(N C Q S T Y)	(A I L F W V)
PtMAP20	Potri.017G144061	20794.00	9.70	177	70.00	21.00	33.00	41.00	49.00
PtMAP20L1	Potri.004G118700	61765.51	9.29	540	45.46	16.99	23.07	26.13	22.53
PtMAP20L2	Potri.017G092100	61279.02	9.18	536	45.45	16.47	22.33	27.14	21.75
PtTPX2-1	Potri.007G138600	92337.81	9.41	817	40.23	14.71	20.22	25.97	24.12
PtTPX2-2	Potri.017G013100	91647.10	9.42	812	41.37	15.15	20.70	24.25	24.46
PtWDL1	Potri.005G055900	40115.68	9.74	365	44.66	13.82	22.48	28.33	20.60
PtWDL2	Potri.008G162800	43127.15	8.66	388	44.32	16.84	19.96	28.45	21.80
PtWDL3	Potri.010G076200	44295.84	8.42	405	42.55	16.11	18.79	26.28	23.44
PtWDL4	Potri.013G042800	39987.47	9.62	362	44.55	15.26	22.98	28.54	21.44
PtWDL5	Potri.006G200400	54009.10	9.71	504	36.01	12.94	19.65	32.06	21.37
PtWDL6	Potri.006G254400	48617.05	8.89	437	41.14	16.66	20.18	26.87	22.43
PtWDL7	Potri.016G066600	46904.30	9.04	504	36.51	13.25	19.42	32.61	21.40
PtWDL8	Potri.018G027500	46799.03	9.90	435	40.57	16.50	20.08	27.36	24.61
PtWDL9	Potri.001G336300	61199.83	9.46	548	39.52	13.89	19.94	28.86	23.80
PtWDL10	Potri.008G129300	71876.15	6.81	646	43.96	17.48	19.00	26.86	22.29
PtWDL11	Potri.008G180900	70957.03	9.55	648	38.47	14.08	20.11	30.82	24.18
PtWDL12	Potri.010G038100	59780.95	8.92	542	42.36	17.39	20.78	25.20	24.00
PtWDL13	Potri.010G053200	58901.13	9.52	534	39.11	13.58	19.44	27.38	26.83
PtWDL14	Potri.010G113000	72081.53	7.63	647	44.14	17.15	19.71	28.49	21.71

According to the properties of amino acids, they are classified as charged amino acids, acidic amino acids, basic amino acids and hydrophobic amino acids. Letters in brackets are amino acids for short.

**Figure 1:**
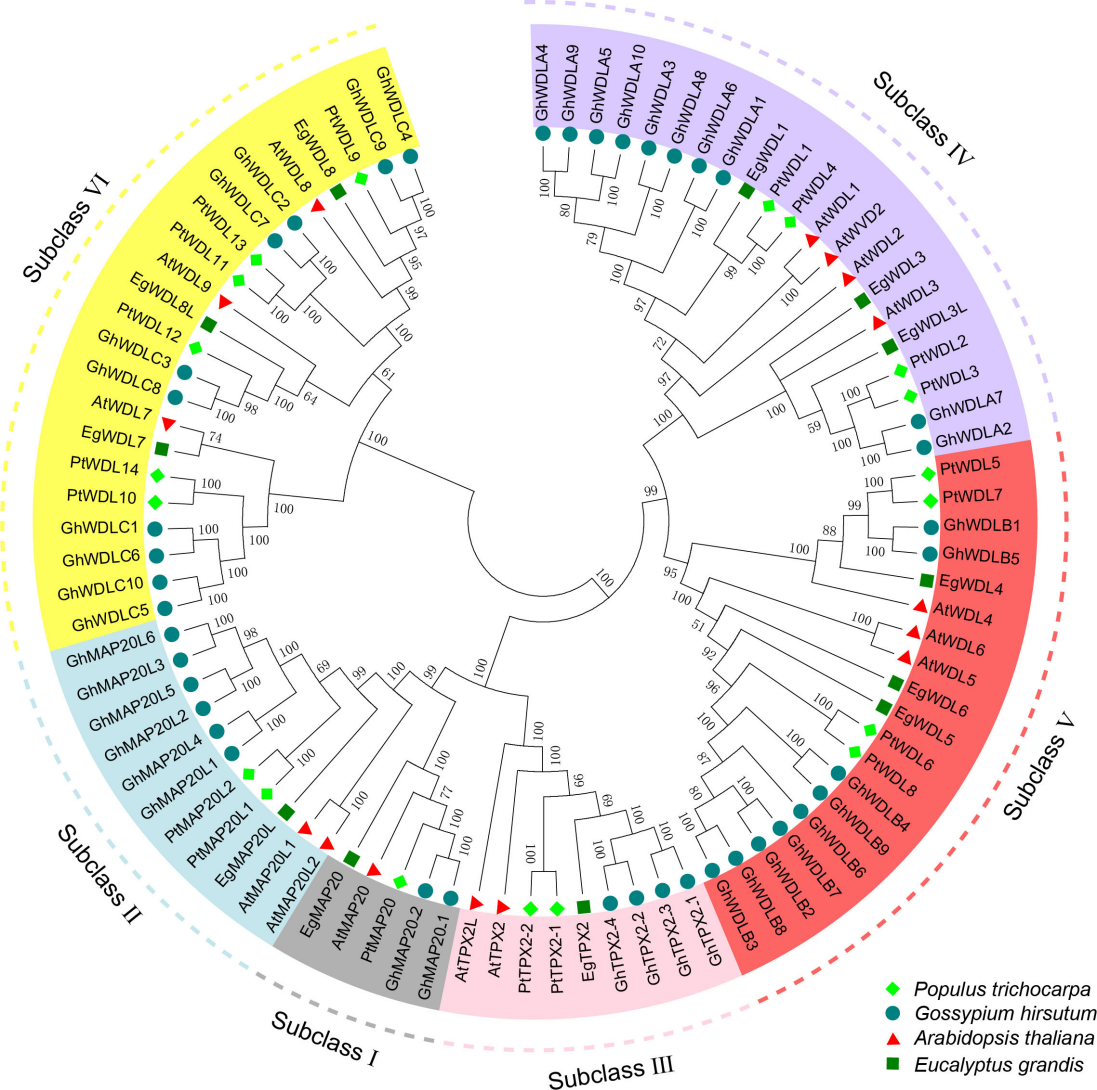
Phylogenetic tree of the PtTPX2 family with AtTPX2, GhTPX2 and EgTPX2 as internal reference. All the proteins were divided into six subclasses (I-VI), with PtTPX2 family members highlighted by bright green diamonds and other species distinguished by different colors and shapes.

To investigate the evolutionary relationships and potential functional characteristics of the PtTPX2 family members, a phylogenetic tree was constructed using all the TPX2 full-length protein sequences from *Populus trichocarpa*, *Eucalyptus grandis*, *Gossypium hirsutum* and *Arabidopsis thaliana* (**Supplementary Table S2**). Based on the homology, the *PtTPX2* genes, together with other *TPX2* genes, were assigned to six different subclasses (Subclass I-VI) (**Figure 1**). Subclass VI contained the largest numbers of *TPX2* genes. Subclass I contained the smallest numbers of *TPX2* genes, including *AtMAP20*, *PtMAP20*, *EgMAP20* and two *GhMAP20* genes. In addition, we examined other evidence to support the reliability of this classification, such as gene structures, conserved motifs (**Supplementary Figure S1**).

### 3.2 Structural analysis of PtTPX2 genes and proteins

**Figure 2:**
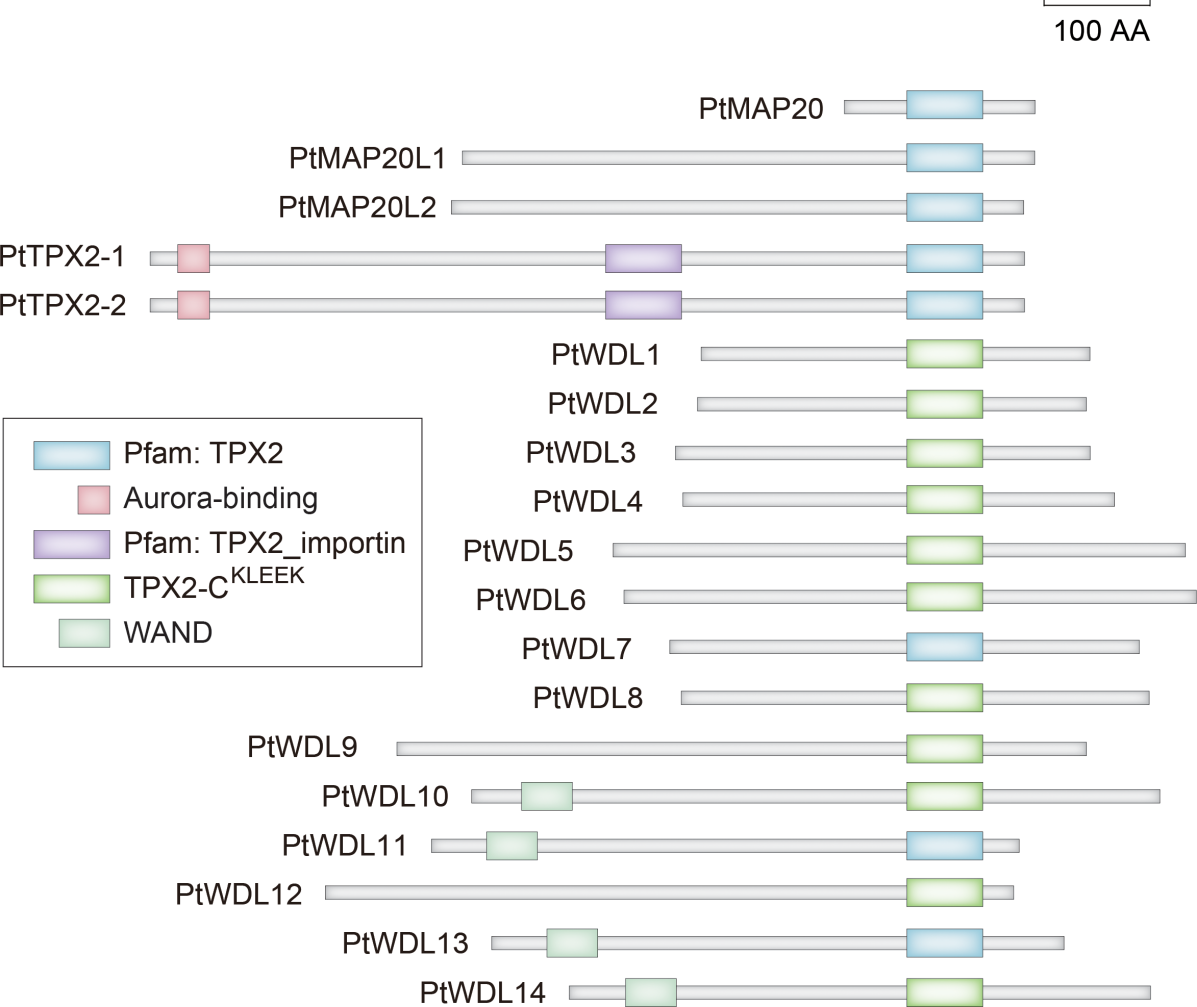
Domain organization of *Populus trichocarpa* TPX2-family proteins.

To further understand the structural diversity and similarity of *PtTPX2* genes and PtTPX2 proteins, we analyzed the exon-intron structures, conserved motifs, and protein domains. Among 19 *PtTPX2* genes, genes grouped in the same subclass had similar gene structures (**Supplementary Figure S1**). The gene with the shortest length, *PtMAP20*, contained an extremely short first exon, and the six *PtWDL* (*PtWDL9*-*PtWDL14*) members also contained a short first exon. However, the subclass II members *PtMAP20L1* and *PtMAP20L2* contained a relatively long first exon among all *PtTPX2* genes. The two genes with the longest lengths, *PtTPX2-1* and *PtTPX2-2*, were comprised of a total of 20/19 exons and 19/18 introns, respectively. The remaining genes generally contained six to nine exons. All 19 TPX2 proteins contain the TPX2 domains (**Figure 2**). In addition, several members have a plant-specific WAND. Among them, PtMAP20, PtMAP20L1, PtMAP20L2, PtTPX2-1 and PtTPX2-2 have the TPX2 domain. All PtWDL proteins have TPX2-C^KLEEK^ domain, except PtWDL7, PtWDL11 and PtWDL13. Only PtTPX2-1 and PtTPX2-2 proteins have TPX2_importin domain and Aurora-binding. PtWDL10, PtWDL11, PtWDL13 and PtWDL14 proteins have WAND domain.

To further analyze the functional regions of PtTPX2 proteins, the conserved motifs were predicted using the MEME program (**Supplementary Figure S1**). After MEME analysis, fifteen conserved motifs were predicted and named as Motif 1 ~ Motif 15. By analyzing the motif distribution of PtTPX2 proteins, the C-terminus of all members contained a well-conserved Motif 1. Most subclass members exhibited a common motif composition. Each subclass had different conserved motifs in the N-terminus regions, such as Motif 12 in subclass II, Motif 10 in subclass III, Motif 6 in subclass IV and Motif 3 in subclass VI, indicating that the specific motif was related to specific biological functions.

### 3.3 Analysis of PtTPX2 proteins’ intrinsically disordered regions

**Figure 3:**
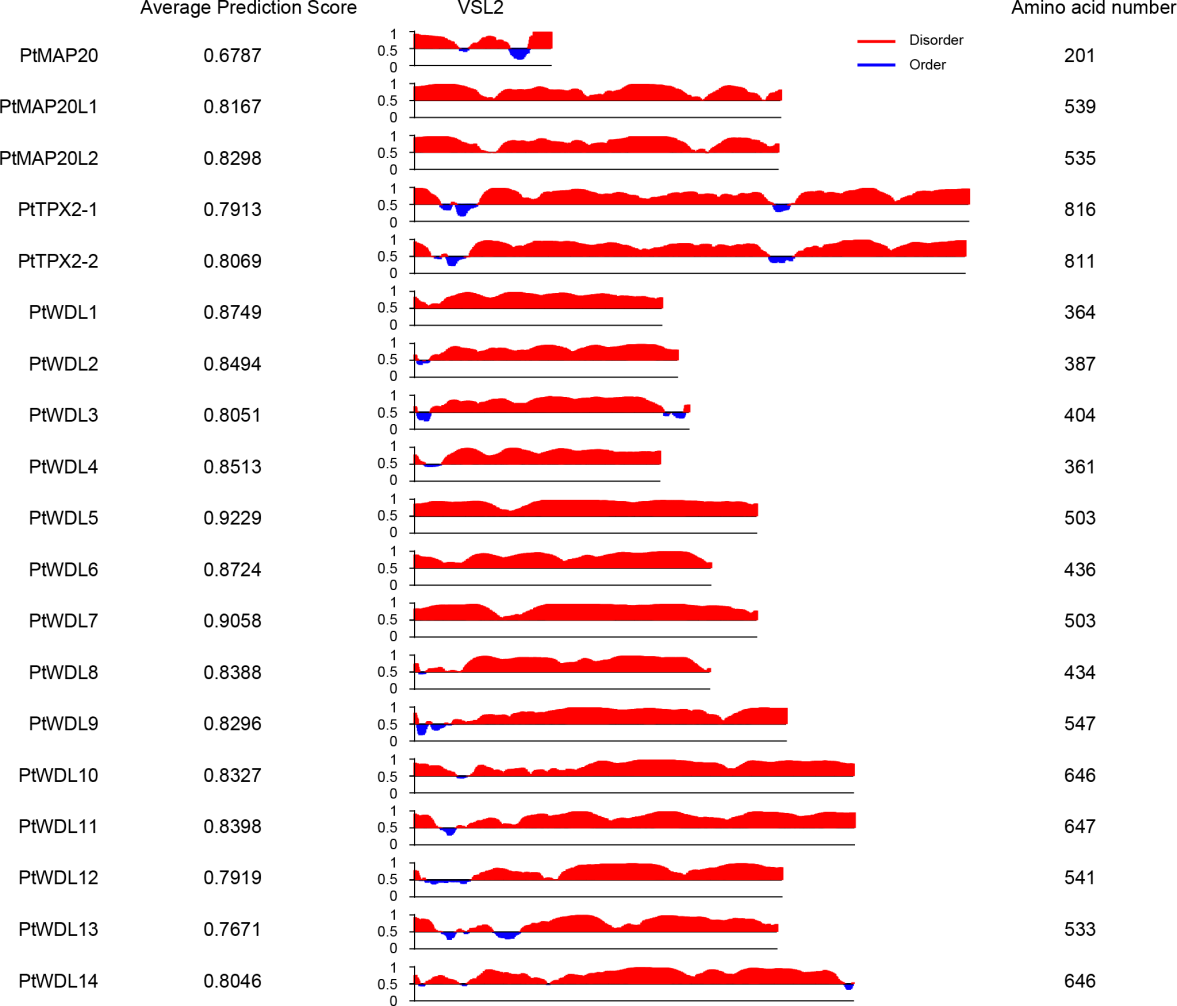
Intrinsically disordered regions prediction of 19 PtTPX2 proteins.

The phase separation of proteins is ubiquitous in cell biology. Multiple MAPs contain a substantial percentage of intrinsically disordered regions (IDRs), which can contribute to protein coacervation and phase separation (Pak et al., 2016). Previous studies have shown that the *Xenopus* TPX2 protein undergoes phase separation to form droplets with tubulin, which is essential for microtubule nucleation (King and Petry, 2020; Safari et al., 2021). Since phase separation often occurs in proteins that have intrinsically disordered regions, we analyzed all PtTPX2 proteins using PONDR program (designed to predict the protein disordered regions) (**Figure 3**). All 19 PtTPX2 proteins were predicted to have disordered regions (average prediction score were greater than 0.5). The PtMAP20L1, PtMAP20L2, PtWDL1, PtWDL5, PtWDL6 and PtWDL7 proteins were highly disordered among all regions. Particularly, the average prediction score of PtWDL5 and PtWDL7 were greater than 0.9. PtTPX2-1 and PtTPX2-2 were both highly disordered in the N- and C-termini. The disordered regions of each protein exhibited high diversity. Based on the above results and the functional reports from previous studies, PtTPX2 proteins may play different biological functions through phase separation under different conditions.

### 3.4 Promoter analysis of PtTPX2 genes

**Figure 4:**
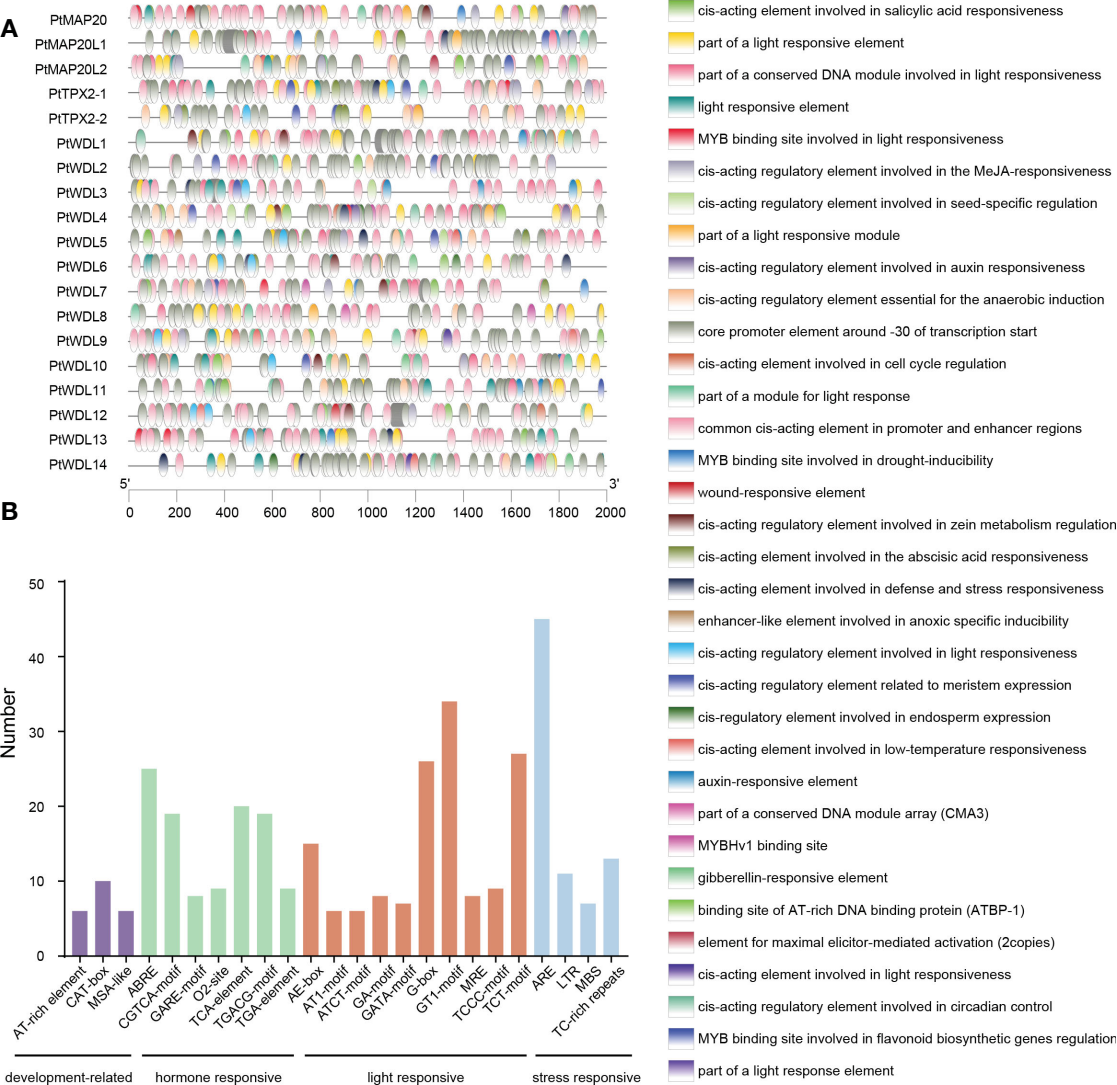
Analysis of *cis*-acting elements in the promoter regions of *PtTPX2* genes. **(A)** Types and distribution locations of *cis*-acting elements in the promoter region of *PtTPX2* gene. **(B)** The number of *cis*-acting elements in the promoter regions of *PtTPX2* genes.

To elucidate the possible regulatory mechanisms of the *PtTPX2* genes in abiotic stress responses, we extracted the 2000 bp sequences upstream of the transcription start site to analyze the *cis*-acting elements (**Supplementary Table S3**). We listed the *cis*-acting elements which are related to development-related, hormone responsive, light responsive and stress responsive in **Figure 4**. Most promoter regions of *PtTPX2* genes contained the Myeloblastosis (MYB) and Myelocytomatosis (MYC) binding sites, which are associated with salt and drought stress responses. In addition, we counted the number of developmental, hormonal, light, and stress-related *cis*-acting elements (**Figure 4**). CAT-box, ABA-responsive element (ABRE), GT1, and AU-rich element (ARE) were the most abundance *cis*-acting elements for development, hormone, light, and stress response, respectively. These results indicated that *PtTPX2* genes is capable of responding to a wide range of abiotic stresses to perform biological functions.

### 3.5 Chromosome distribution and collinear analysis

**Figure 5:**
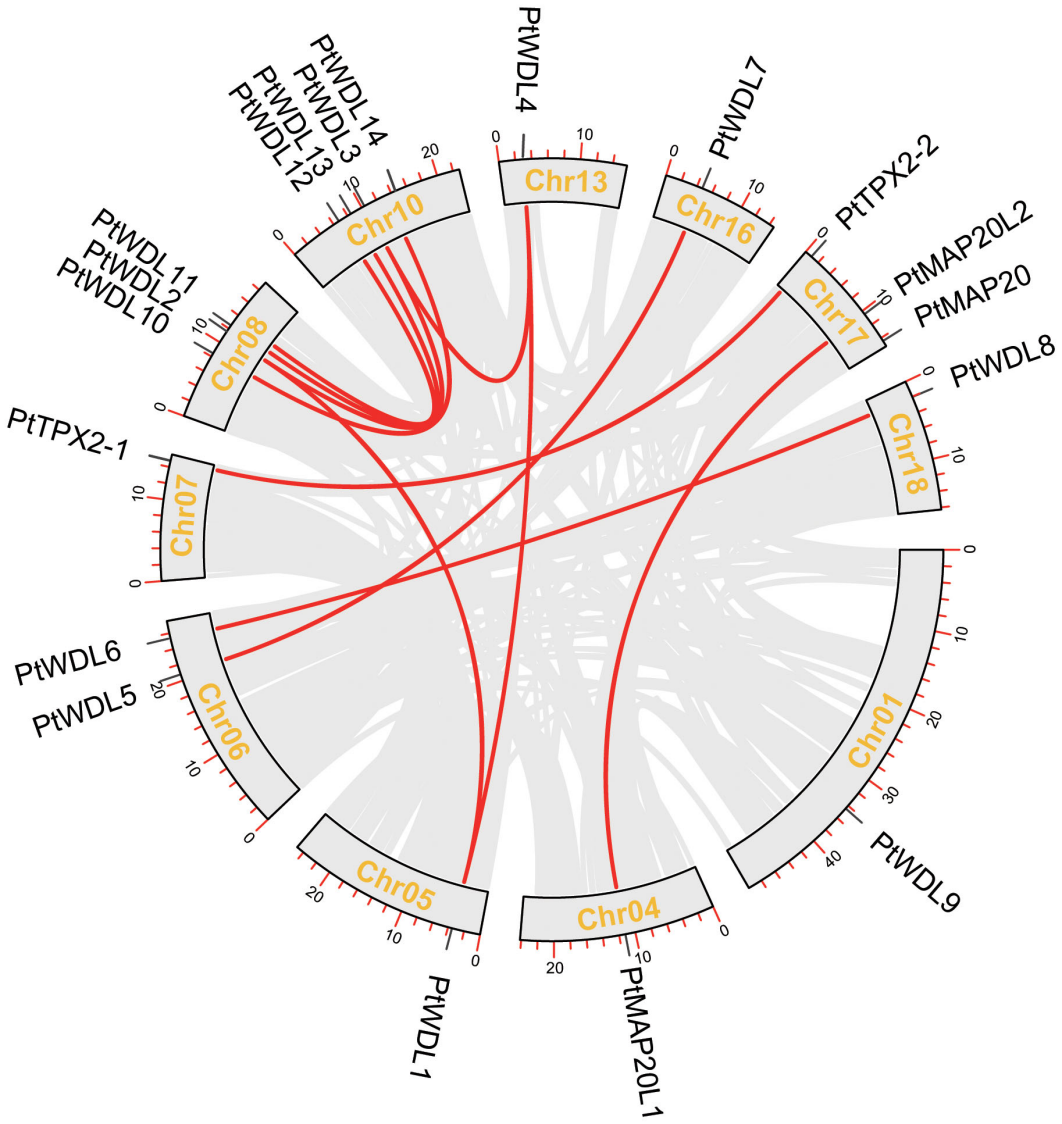
Relationship between different *PtTPX2* genes in different chromosomes. Grey lines indicate all homologous blocks in the *Populus trichocarpa* genome, and the red lines indicate duplicated *PtTPX2* gene pairs.

**Figure 6:**
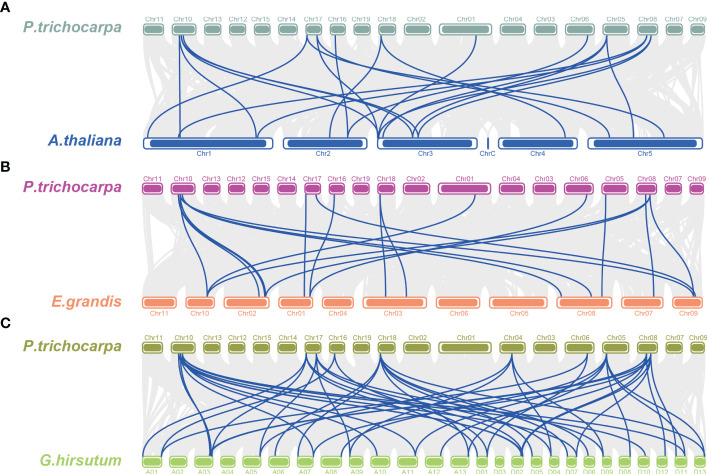
Collinearity analysis between different genomes. **(A)** Collinear analysis of *Populus trichocarpa* and *Arabidopsis thaliana*. **(B)** Collinear analysis of *Populus trichocarpa* and *Eucalyptus grandis*. **(C)** Collinear analysis of *Populus trichocarpa* and *Gossypium hirsutum*. Grey lines indicate the collinear blocks within *Populus trichocarpa* and different genomes, the blue line indicates collinear gene pairs.

The chromosome distribution of the *PtTPX2* genes was mapped using the TBtools software based on annotated information on the genome structure of *Populus trichocarpa*, *Arabidopsis thaliana*, *Gossypium hirsutum* and *Eucalyptus grandis*. The 19 *PtTPX2* genes were unevenly distributed on 11 chromosomes of *Populus trichocarpa* (**Figure 5**), indicating the diversification and complexity of the TPX2 family. Chr10 contained four *TPX2* genes, three of which belonged to Subclass VI. Chr08 and Chr17 both contained three *TPX2* genes. Chr06 contained two *TPX2* genes, both of which belonged to Subclass V. Chr01, Chr04, Chr05, Chr07, Chr13, Chr16 and Chr18 each contained one *TPX2* gene. Gene duplication events were detected by MCScanX, and tandem duplicated genes were not detected. Collinearity analysis showed that all *PtTPX2* had at least one collinear gene, except *PtWDL9* and *PtMAP20* (**Figure 5**). In order to further analyze the homology and collinearity of *TPX2* genes in different species, we also performed an interspecies collinearity analysis using the Multiple Synteny Plot function of TBtools software. A comparison of the collinear genomic blocks of *Populus trichocarpa* with *Arabidopsis thaliana*, *Gossypium hirsutum* and *Eucalyptus grandis*, showed that several *PtTPX2* genes were homologous (**Figure 6**) owing to chromosomal segmental duplication. *Populus trichocarpa* and *Gossypium hirsutum* were more collinear gene pairs. This conclusion suggests that *Populus trichocarpa* was more closely related to *Gossypium hirsutum*. This maybe attributable to that *Populus trichocarpa* and *Gossypium hirsutum* grow in similar environments. These results suggested that segmental duplication was essential in generating of the *PtTPX2* genes family in *Populus trichocarpa*.

### 3.6 Expression profiles of PtTPX2 genes in different plant tissues

**Figure 7:**
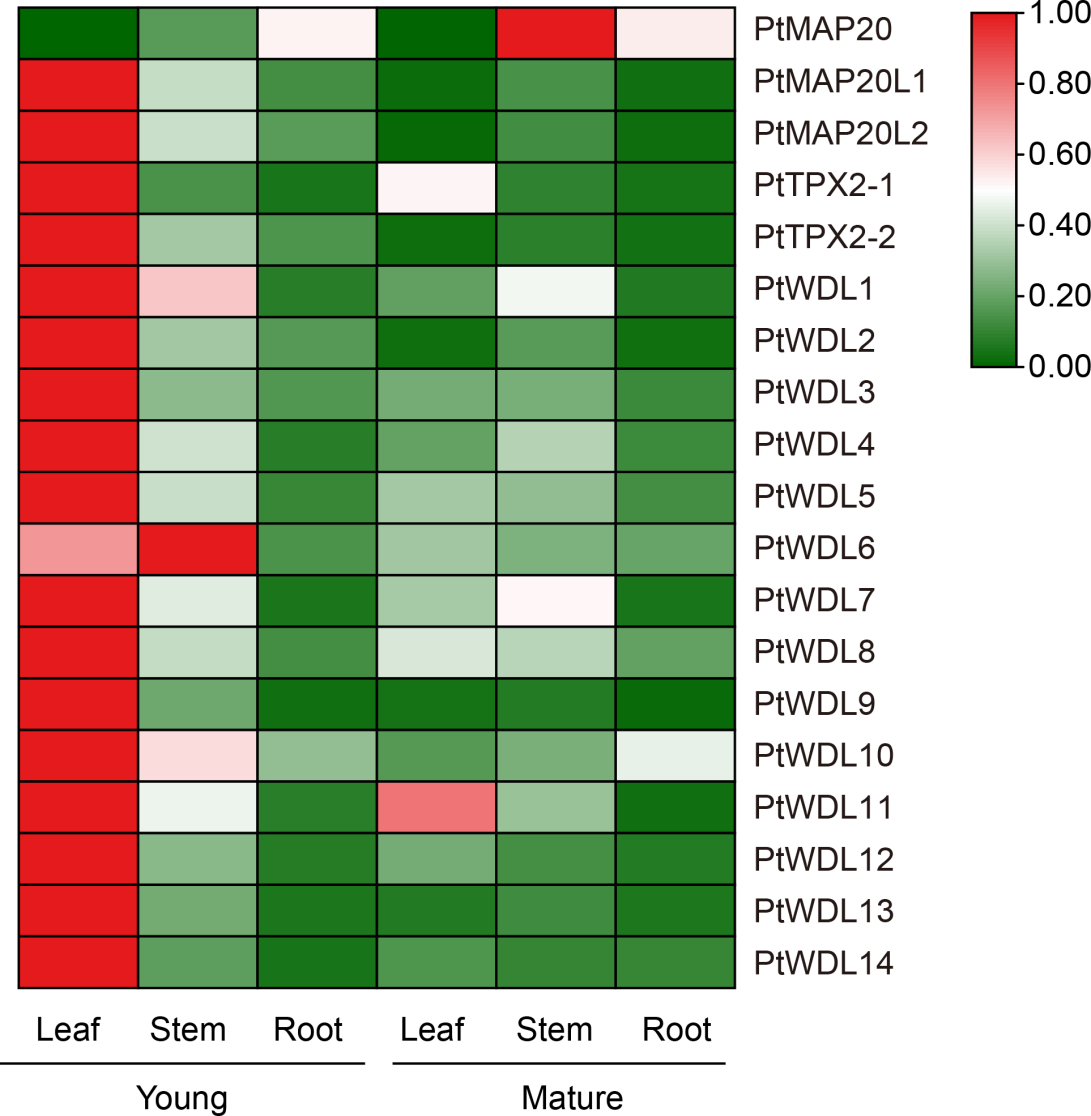
Heatmap of *PtTPX2* gene expression patterns in different growing periods, young and mature. Green represents lower gene expression levels and red represents higher gene expression levels. The color represents the level of expression of each gene in each sample. The gene expression levels were calculated based on 2^− ΔΔCt^.

Tissue-specific expression analysis showed that *PtTPX2* gene expression levels were significantly different in different tissues, but almost all *PtTPX2* genes had low expression levels in mature leaves, stems and roots (**Figure 7**; **Supplementary Table S4**). There was obvious clustering among different subclass members of *PtTPX2*, and *PtMAP20*, belonging to the Subclass I, was significantly different from other genes. The expression levels and trends of *PtMAP20L1* and *PtMAP20L2* in different tissues were basically consistent, they belong to Subclass II. The expression of all *PtWDL* genes in young leaves was significantly higher than that in other tissues. The expression of *PtMAP20* gene showed the highest trend in mature stems, but it was low expressed in young leaves. *PtWDL11* also showed high expression in mature leaves, but the expression level was still lower than the young leaves. In addition, *PtWDL1* and *PtWDL10* were also highly expressed in young stems, but significantly lower than their expression in young leaves. Notably, the expression of *PtWDL6* is highly expressed in both young leaves and young stems and the expression level in young stems is higher than that in other tissues, whereas *PtWDL5*, *PtWDL7* and *PtWDL8*, its closest homolog, were highly expressed only in the young leaves.

### 3.7 Analysis of PtTPX2 genes based on RT-qPCR under salt stress

**Figure 8:**
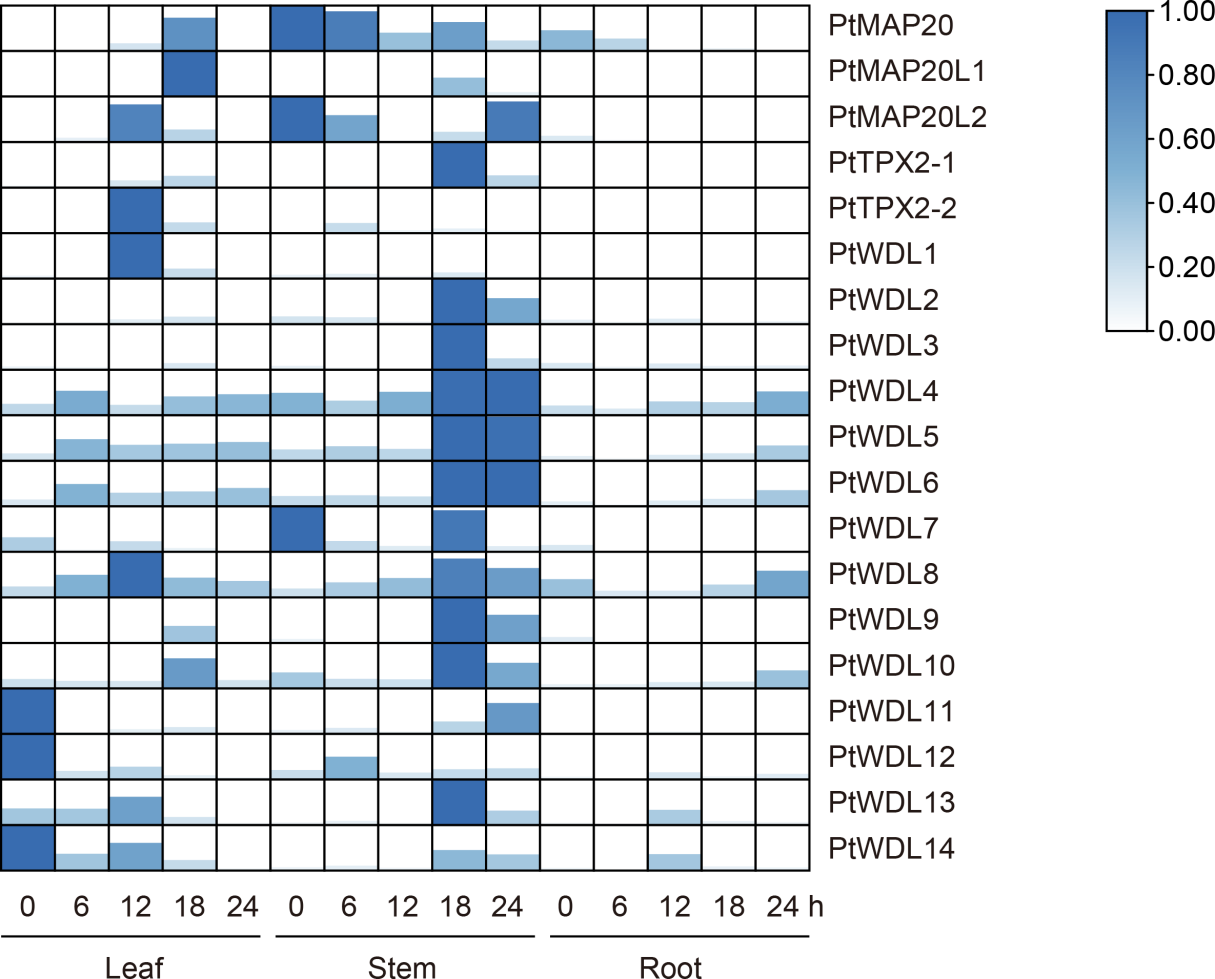
Heatmap of *PtTPX2* gene expression patterns in different tissues and processing time under salt stress. The graphic size and color represent the level of expression of each gene in each sample. The gene expression levels were calculated based on 2^− ΔΔCt^.

Under salt stress, *PtWDL* genes in stems showed an up-regulation phenomenon with the increase of treatment time, except for *PtWDL1* and *PtWDL12*. The expression of *PtWDL11*, *PtWDL12* and *PtWDL14* genes showed a decreasing trend in leaves. Notably, *PtWDL11*, *PtWDL12* and *PtWDL14* genes all belong to Subclass VI. Moreover, these genes were all expressed without salt stress, suggesting that they may be involved in the regulation of poplar growth. The expressions of *PtMAP20* and *PtMAP20L1* showed an increasing trend in leaves and reached the highest level in 18 h. Additionally, the expressions of *PtMAP20L2*, *PtTPX2-1* and *PtTPX2-2* all increased first and then decreased, the expression levels of them peaked after 12 h of salt stress (**Figure 8**) (**Supplementary Table S4**), suggesting that they might have similar functions throughout the salt stress period. The results showed that the expression levels of all *PtWDL* genes increased significantly in the late stage of salt stress, suggesting that they may be more involved in the regulation activities in the late stage of salt stress.

### 3.8 Analysis of PtTPX2 genes based on RT-qPCR under heat stress

**Figure 9:**
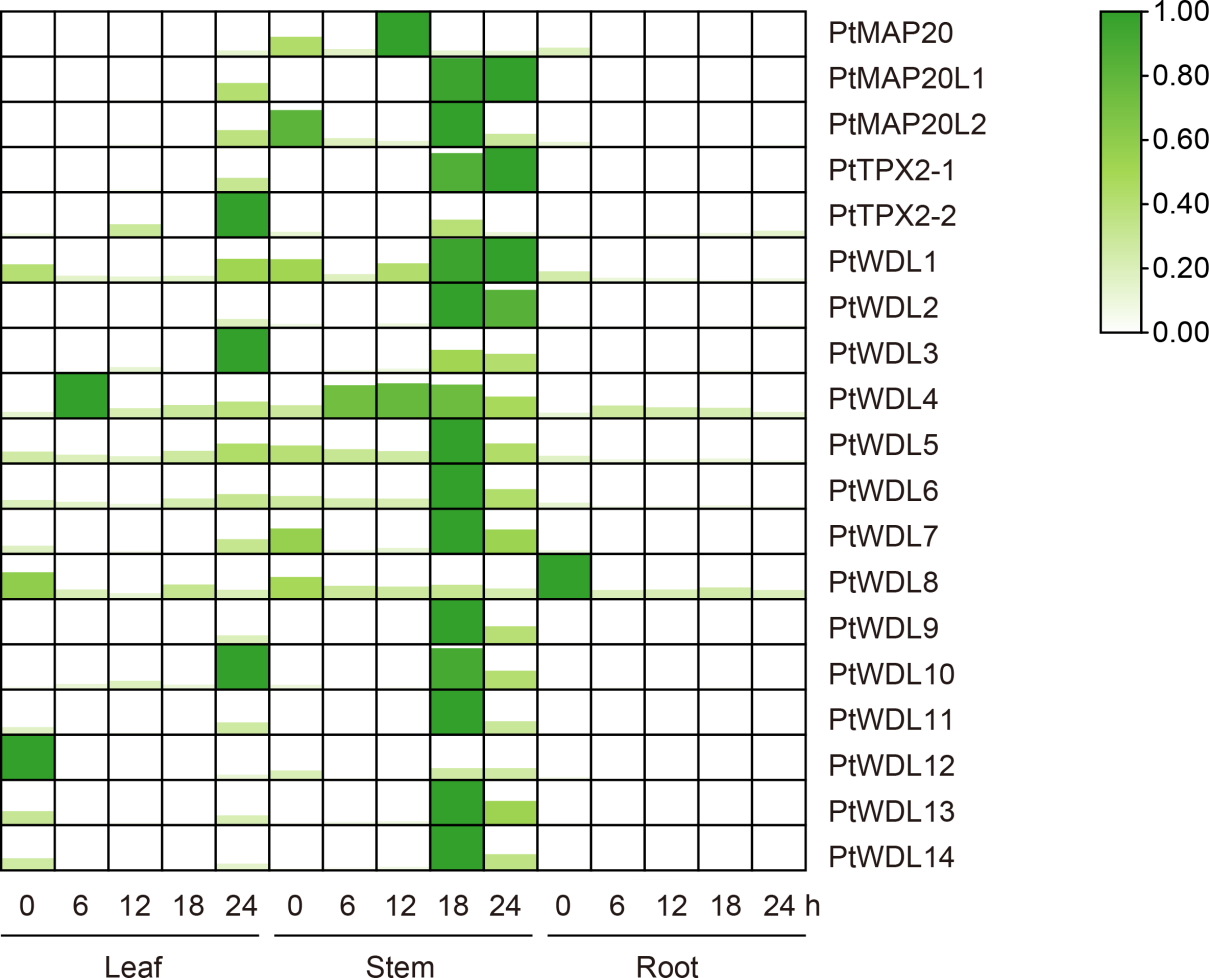
Heatmap of *PtTPX2* gene expression patterns in different tissues and processing time under heat stress. The graphic size and color represent the level of expression of each gene in each sample. The gene expression levels were calculated based on 2^− ΔΔCt^.

The present study results showed that the expression profiles of *PtTPX2* showed genes different responses to artificially simulated temperature (42°C) stress for different durations. Moreover, the expression profiles of *PtTPX2* also showed significant differences among different tissues. Under heat stress, all *PtTPX2* genes have the most obvious changes in leaves and stems, which indicates that *PtTPX2* genes directly respond to heat stress and play biological functions in the above-ground parts of the plant. The present study results showed that most of the *PtTPX2* genes had the highest expression at 18 h after heat stress in the stems. However, several genes are different. *PtMAP20*, belong to Subclass I, was peaked in 12 h after heat stress in the stems. *PtTPX2-1* and *PtWDL1* were peaked in 24 h after heat stress in the stems. *PtTPX2-2*, *PtWDL3* and *PtWDL10* were peaked in 24 h after heat stress in the leaves. These results indicate that these genes play a regulatory role in the late stage of heat stress. *PtWDL4*, belong to Subclass IV, was peaked in 6 h after heat stress in the leaves, suggesting that it can play a regulatory role in the early stage of heat stress, which also proves that the leaf organs of plants are crucial for the perception of heat stress. In addition, the expression of *PtWDL8* and *PtWDL12* did not change significantly in stems. *PtWDL8* and *PtWDL12* was highly expressed in roots and leaves without heat stress, but could not be detected after heat stress (**Figure 9**). These results indicate that *PtTPX2* genes mostly respond to heat stress in the stems.

### 3.9 Analysis of PtTPX2 genes based on RT-qPCR under drought stress

**Figure 10:**
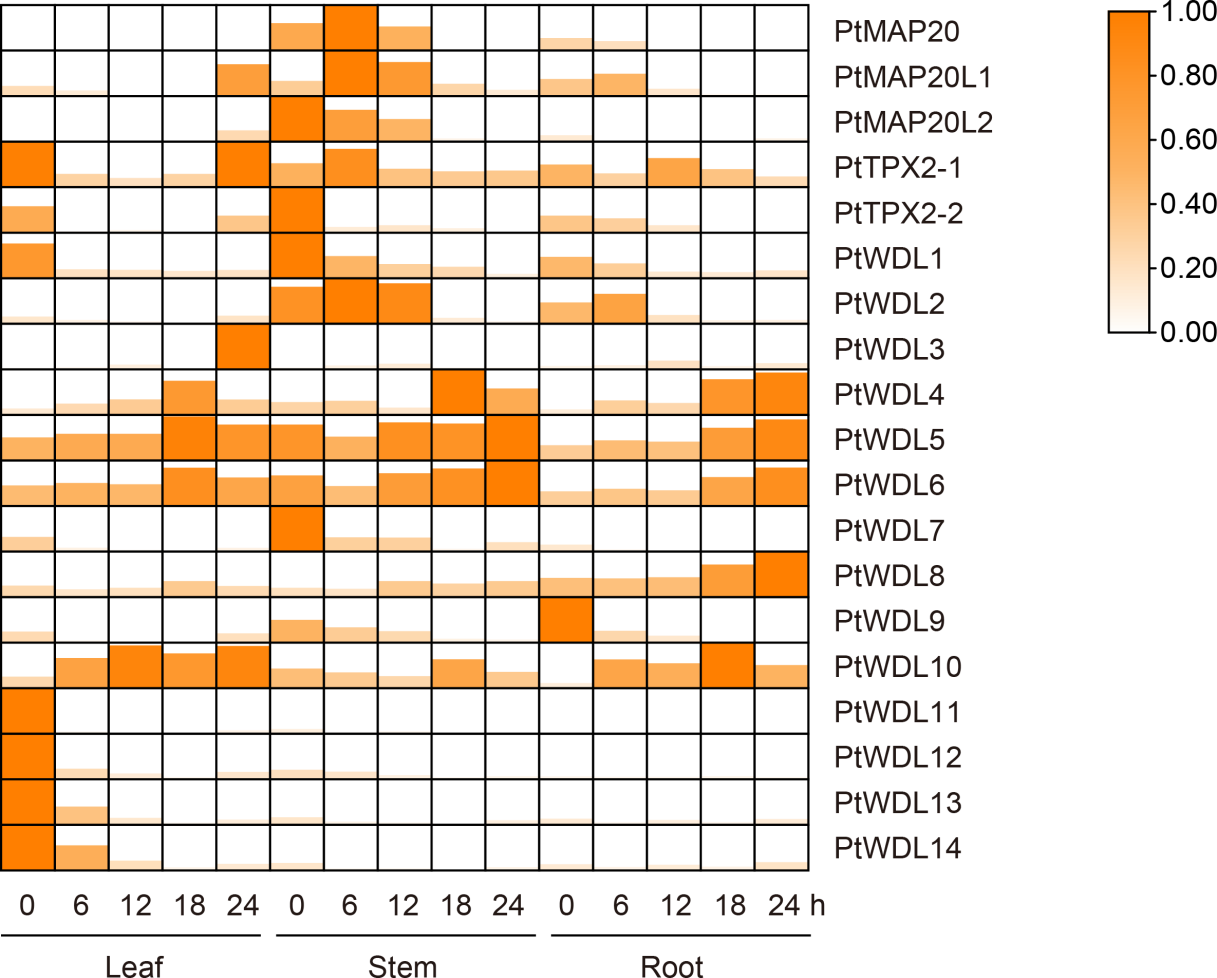
Heatmap of *PtTPX2* gene expression patterns in different tissues and processing time under drought stress. The graphic size and color represent the level of expression of each gene in each sample. The gene expression levels were calculated based on 2^− ΔΔCt^.

*PtWDL4*, *PtWDL5* and *PtWDL6*, belong to Subclass IV and V, were up-regulated in leaves, roots and stems tissues over time under drought stress, suggesting that these genes may play a key role in poplar’s specific response to drought stress. Furthermore, *PtMAP20*, *PtMAP20L1* and *PtMAP20L2* were only significantly expressed in stems. The expression of *PtMAP20* and *PtMAP20L1* reached a peak at the 6 h of drought treatment, and then decreased. These results suggest that these genes may play a role in the early stages of drought stress. However, *PtMAP20L2*, belong to Subclass II with *PtMAP20L1*, was a high expression level without drought stress, suggesting that the growth of poplar itself is regulated by *PtMAP20L2*. Notably, *PtWDL1* and *PtWDL7* may have similar biological functions to *PtMAP20L2*. In addition, the expressions of *PtWDL11*, *PtWDL12*, *PtWDL13* and *PtWDL14* were only significantly down-regulated in leaves. Moreover, these genes peaked without drought stress, suggesting that these genes might not be regulated by drought stress (**Figure 10**). These results suggest that *PtTPX2* genes differently respond to drought stress, however, these genes in the same subclass mostly have similar biological functions.

### 3.10 Subcellular localization analysis of PtWDL6

**Figure 11:**
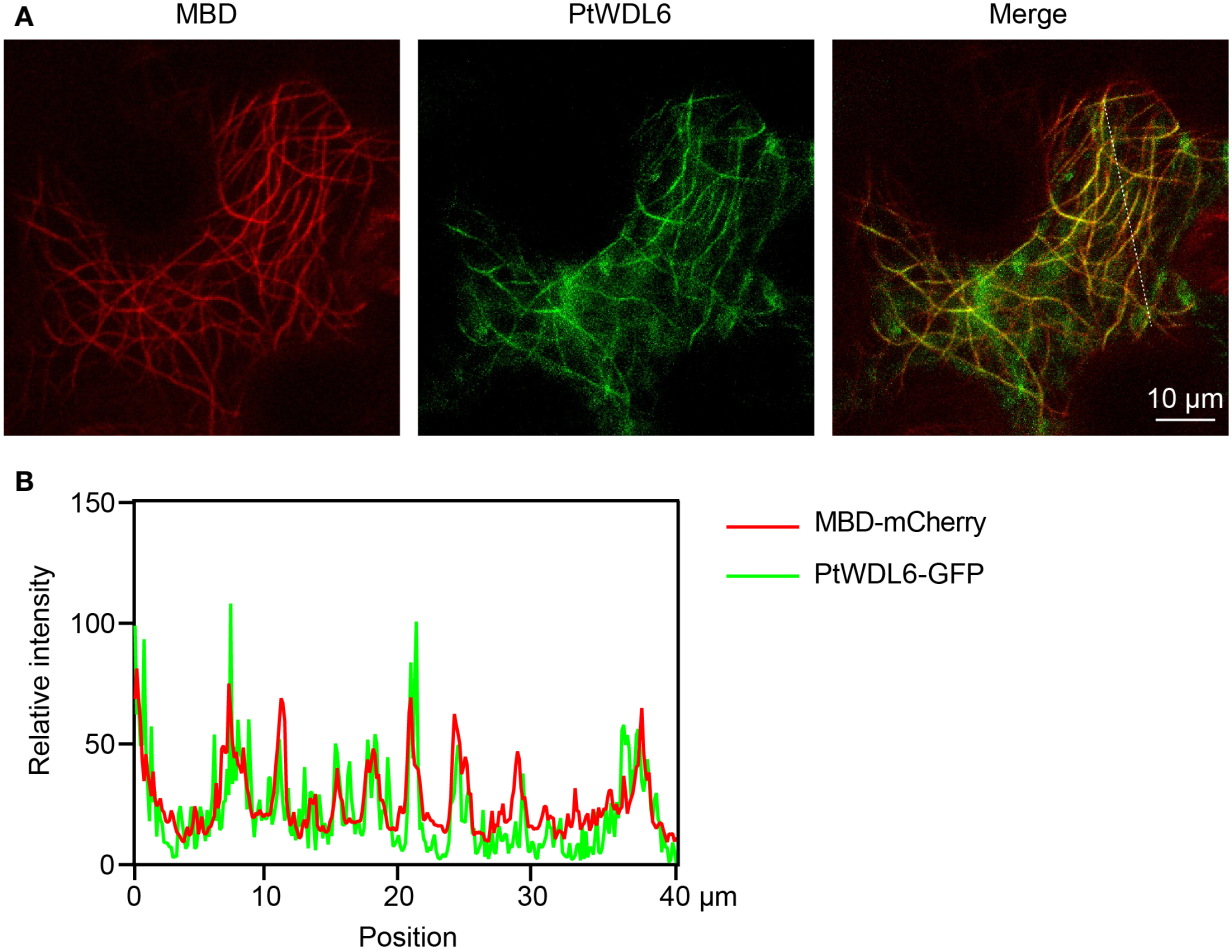
Subcellular localization of PtWDL6 proteins. **(A)** PtWDL6 colocalizes with cortical MTs detected in tobacco cells. **(B)** Plot of a line scan drawn showing a strong correlation between the spatial localization of PtWDL6 and cortical MTs.

Based on the above results, *PtWDL6* is significantly respond to salt, drought and heat stresses. Therefore, *PtWDL6* was cloned and studied in the following work. PtWDL6-GFP and MBD-mCherry (labeled MTs) were temporarily expressed in tobacco leaf epidermal cells and visualized by a confocal laser-scanning microscope (**Figure 11**). As shown in **Figure 11**, the PtWDL6-GFP fluorescence overlapped with the red fluorescent signal of cortical MTs. Colocalization was analyzed by plotting the signal intensities of PtWDL6-GFP and MTs using ImageJ software (**Figure 11**). As demonstrated, PtWDL6 localized to MTs in the tobacco cells.

## 4 Discussion

The microtubule is a dynamic and adaptive structure, and its functions and dynamics depend on regulation by microtubule-associated proteins (Dixit and Cyr, 2004; Ehrhardt and Shaw, 2006; Akhmanova and Steinmetz, 2010; Hamant et al., 2019). TPX2 is a class of conserved microtubule-associated proteins which regulate microtubule dynamics in response to diverse developmental and environmental cues (Wittmann et al., 2000; Neumayer et al., 2014; Wadsworth, 2015). Poplar has a high economic value and serves as a model for elucidating molecular mechanisms of development and stress tolerance in tree species. However, genome-wide analyses of the *TPX2* genes of *Populus trichocarpa* have not been performed, and the regulatory functions of *PtTPX2* genes involved in abiotic stress responses remain unclear. In the present study, 19 *PtTPX2* genes were screened and systematically analyzed. The phylogenetic analysis was conducted with four plants species: *Populus trichocarpa*, *Eucalyptus grandis*, *Gossypium hirsutum* and *Arabidopsis thaliana*. The TPX2 protein family in *Populus trichocarpa* was divided into six subclasses to further identifying the protein conservation. The similar arrangements of gene structures and conserved motifs in the same subclass further prove the correctness of the *TPX2* classifications. All *TPX2* members contained well-conserved motif 1 in the C-terminus regions, and subclass members had different conserved motifs in the N-terminus regions, suggesting that *TPX2* genes may have different biological functions.

Under abiotic stresses, plants usually activate various mechanisms to resist the adverse environment (Chai et al., 2016). Soil salinization is one of the main factors affecting the productivity of forestry (Yang et al., 2018; Litalien and Zeeb, 2020; Van Zelm et al., 2020). Under high-salinity conditions, excessive cytosolic Na^+^ breaks the dynamic balance of basic mineral nutrients that limit the growth and development of trees. In addition, multiple studies have demonstrated that many other regulatory factors and molecular mechanisms are involved in the process by which plants resist salt stress (Osakabe et al., 2014; Zhao et al., 2018; Wei et al., 2022; Deng et al., 2023). Heat stress is one of the important ecological factors affecting plant physiological processes. In recent years, with the impact of climate change such as the increase in global average temperature, the phenomenon of plants suffering from heat stress has become more and more frequent (Hassan et al., 2021). Plants respond to heat stress mainly through the activation of subsequent molecular cascades associated with heat shock factors and their main targets (heat shock proteins), to cellular responses and then to the plant’s own phenotypic response against heat stress (Kang et al., 2022). Drought stress seriously affects plant growth, development, reproduction and other life activities. It also affects plant morphology, structure and physiological function. Changes in plant biomass, water use efficiency, photosynthetic system, osmotic regulation ability, cell membrane stability, antioxidant system defense ability and hormone levels are often used to judge the ability of plants to resist drought stress (Kuromori et al., 2022). In this study, *PtTPX2* gene was differentially expressed in response to salt, heat and drought stresses. In addition, *TPX2* genes in different plant tissues were also responsive to cold and osmotic stresses (Kumar et al., 2016).

The microtubule cytoskeleton controls plant growth and cell morphogenesis, as well as participates in the adaptation of plants to stress. Cortical MTs reorganized new microtubule networks to aid plant survival under stress. Several MAPs have been implicated in these processes (Wang et al., 2011; Zhang et al., 2012; Li et al., 2017). Previous studies have shown that some upstream transcription factors regulate the expression of the *AtTPX2* gene (Sun et al., 2015; Dou et al., 2018). Therefore, analyzing the promoter region of genes is conducive to screening candidate upstream regulatory factors. In this study, numerous *cis*-acting elements were found in promoters, and they are closely associated with various abiotic stresses. The promoter region of *PtTPX2* contained a large number of binding elements, which are also related to the stress response (Major et al., 2017; Guo et al., 2022). The results of RT-qPCR showed that salt stress, heat stress and drought stress can significantly change the *PtTPX2* gene in different tissues or treatment times. This indicated that *PtTPX2* gene had a positive response to stress treatment (**Figure 8**–**10**). In addition, subcellular localization showed that *PtWDL6* was co-localized with MTs in plant cells (**Figure 11**). That is, *PtWDL6* may respond to abiotic stress by regulating MTs. Therefore, *TPX2* gene may be involved in the regulation of MTs to help plants resist abiotic stresses and adapt to the environment.

## 5 Conclusions

Collectively, 19 *Populus trichocarpa TPX2* genes were comprehensively analyzed, including gene structures, phylogenetic characteristics, intrinsically disordered regions, *cis*-regulatory elements, expression patterns and functional characterizations. Through RT-qPCR data mining, we found that *PtTPX2* gene was significantly differentially expressed under various abiotic stresses. Meanwhile, *PtWDL6* was cloned and co-localized with cortical MTs in tobacco cells. Taken together, these findings provide important information for the elucidation of the function of *PtTPX2* genes and establish a foundation for future studies on the role of *TPX2* genes in stress tolerance.

## Data availability statement

The original contributions presented in this study are included in the article/**Supplementary Materials**. Further inquiries can be directed to the corresponding author.

## Author contributions

NLian conceived and designed the study. NLian, MQ, SW and NLi conducted the experiments and wrote the manuscript. MQ and NLi analyzed the data. SW and LL assisted with the experiments. LL, YZ and JX provided guidance on experimental methods. JW provided the analysis method used for the experimental process. RW revised the manuscript. All authors contributed to the article and approved the submitted version.

## Supplementary material

Supplementary Figure 1 Structure of *PtTPX2* genes and motif composition of PtTPX2 proteins. (UTR: Untranslated Regions. CDS: Coding DNA Sequence).

Supplementary Table 1 qPCR Primers.

Supplementary Table 2 Analysis of the location of *TPX2* in the chromosome.

Supplementary Table 3 Promoter *cis*-acting element analysis.

Supplementary Table 4
*PtTPX2* gene expression patterns.
