# Supplementary figures and images for: Genome-wide analysis of TPX2 gene family in Populus trichocarpa and its specific response genes under various abiotic stresses
Source: Front Plant Sci. 2023 Mar 13;14:1159181. doi: 10.3389/fpls.2023.1159181 (PMC10040543; doi:10.3389/fpls.2023.1159181)

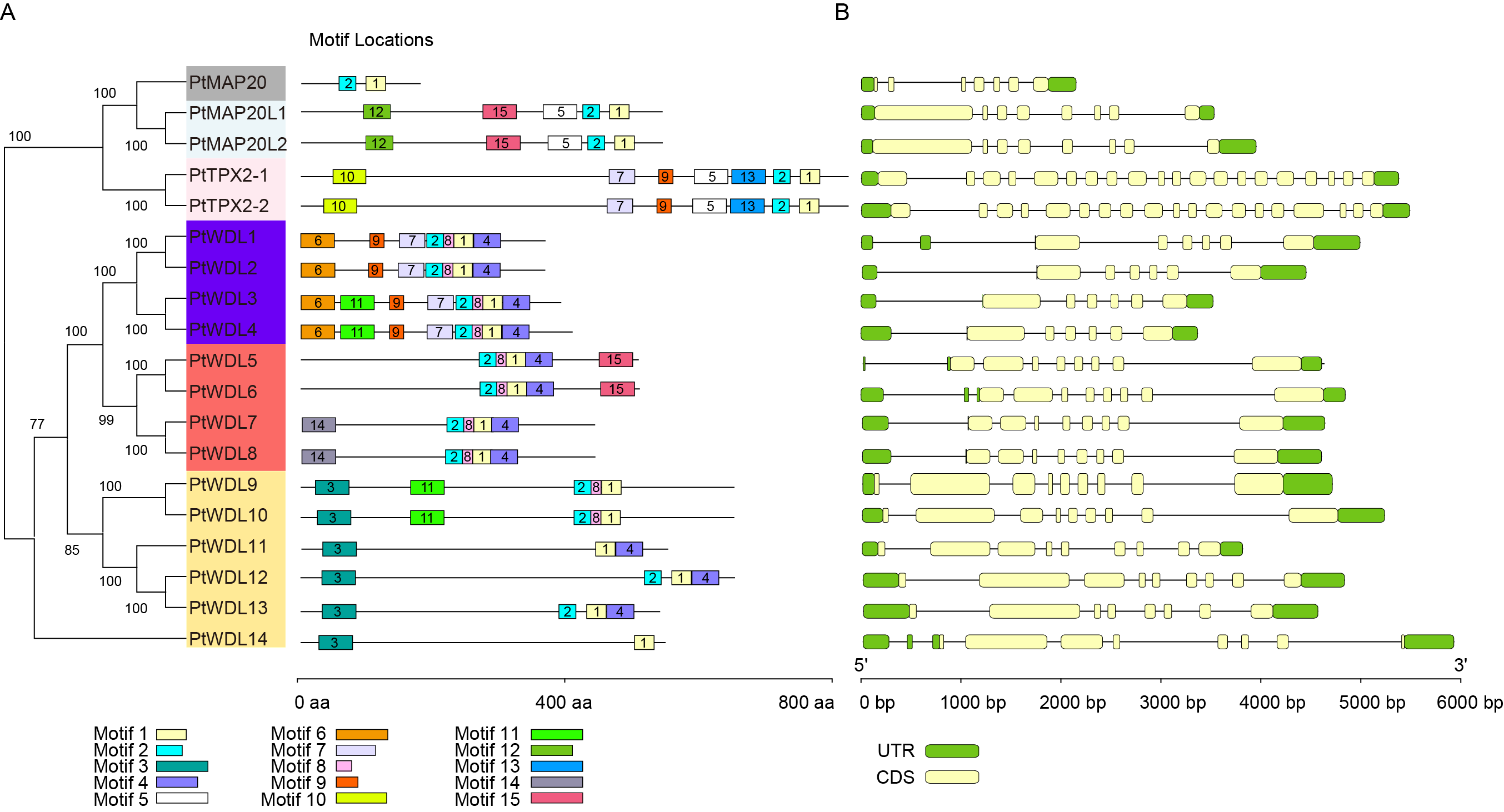

Supplement: Supplementary Figure 1 — Structure of PtTPX2 genes and motif composition of PtTPX2 proteins. (UTR: Untranslated Regions. CDS: Coding DNA Sequence). [file DataSheet_1.zip › Supplementary materials/Figure S1.tif]
